# Comparative Pharmacokinetics and Safety of Orally Disintegrating and Conventional Tablet Formulations of Zastaprazan in Healthy Subjects

**DOI:** 10.1002/prp2.70337

**Published:** 2026-10-16

**Authors:** Hyung‐Gun Kim, Seunghyun Kang, Sunyoung Min, Heejoo Hong, Moonjung Bae

**Affiliations:** ^1^ Biomedical Research Institute, H+ Yangji Hospital Seoul Republic of Korea

**Keywords:** drug safety, easy to take anywhere, orally disintegrating tablet, pharmacokinetics, potassium‐competitive acid blocker, zastaprazan

## Abstract

Zastaprazan, a potassium‐competitive acid blocker, has been developed as an orally disintegrating tablet (ODT) to improve administration flexibility. This study evaluated the pharmacokinetics and safety of a single 20 mg dose of Zastaprazan ODT administered with or without water compared with the conventional tablet in healthy volunteers. In this open‐label, randomized, single‐dose, 6‐sequence, 3‐period crossover study, 54 healthy volunteers received a conventional tablet with water, an ODT with water, and an ODT without water. Serial blood samples were collected up to 36 h post‐dose, and pharmacokinetic parameters were calculated using a non‐compartmental method. Safety was assessed through adverse events, laboratory tests, vital signs, and electrocardiograms. Fifty‐one subjects completed all periods. The geometric mean ratios (90% confidence intervals) of *C*_max_ and AUC_t_ were 95.05% (88.38%–102.21%) and 108.00% (100.50%–116.06%) for ODT without water versus the conventional tablet, and 90.85% (84.96%–97.16%) and 101.88% (96.69%–107.36%) for ODT with water versus the conventional tablet, respectively. All 90% confidence intervals were within the predefined bioequivalence range. *K*_a_ and *T*_max_ showed similar patterns across treatments, and no apparent additional early systemic exposure was observed within 30 min following ODT administration. Two mild adverse events were reported in one subject, with no serious adverse events. Zastaprazan ODT administered either with or without water was pharmacokinetically equivalent to the conventional tablet, with no apparent additional early systemic exposure and no clinically meaningful safety concerns.

**Trial Registration:** ClinicalTrials.gov: NCT07181538

**Abbreviations**
AUC: area under the plasma concentration–time curve
AUC_∞_
: area under the plasma drug concentration–time curve from time zero to infinity
AUC_t_
: area under the plasma concentration–time curve from time zero to last measurable concentration
CL/F: apparent clearance
*C*_max_
: maximum plasma concentration
GMR: geometric mean ratios
LC–MS/MS: liquid chromatography–tandem mass spectrometry
ODT: orally disintegrating tablet
*t*_1/2_
: terminal elimination half‐life
*T*_max_
: time to reach maximum plasma concentration
*V*_d_/*F*
: apparent volume of distribution

## 1 Introduction

Acid‐related disorders, including reflux esophagitis and peptic ulcer disease, are commonly encountered in clinical practice [1, 2, 3]. The management of acid‐related disorders involves pharmacological therapy in conjunction with lifestyle modifications, such as dietary regulation and adjustment of physical activity. Proton pump inhibitors (PPIs), which suppress gastric acid secretion, are widely used as the standard therapeutic strategy for acid suppression [4, 5].

More recently, potassium‐competitive acid blockers (P‐CABs), which provide more rapid, potent, and sustained suppression of gastric acid secretion compared with conventional PPIs, have emerged as a novel therapeutic alternative [6, 7, 8]. P‐CABs can be administered irrespective of meal timing, thereby potentially improving treatment adherence, and their sustained acid‐inhibitory effect enables more effective control of symptoms such as nocturnal acid reflux [9].

Zastaprazan is a novel potassium‐competitive acid blocker developed in Korea, designed to achieve a rapid onset of action and sustained suppression of gastric acid secretion. In clinical studies conducted in healthy volunteers, zastaprazan demonstrated favorable safety and tolerability profiles and was shown to produce rapid and potent inhibition of gastric acid secretion [10]. Furthermore, in comparative studies against conventional proton pump inhibitor therapy, zastaprazan was demonstrated to be non‐inferior in terms of the cumulative healing rates at 8 weeks for erosive esophagitis and gastric ulcer [11, 12]. Taken together, these findings suggest that zastaprazan may represent an appropriate therapeutic option among P‐CAB agents for the management of acid‐related disorders.

Zastaprazan is currently available as a conventional tablet formulation. However, conventional oral tablets may be inconvenient for certain patient populations, including older individuals, patients with dysphagia, or those in situations where water intake is restricted, which may in turn lead to reduced treatment adherence. To address these limitations, an orally disintegrating tablet (ODT) formulation of zastaprazan, which can be administered with or without water and is convenient to take anywhere, has been developed by Onconic Therapeutics, a subsidiary of Jeil Pharmaceutical Co. Ltd., in Korea. This ODT formulation contains an orange‐flavored excipient (Orange Micron) and was designed to enhance palatability and maximize patient adherence.

Orally disintegrating tablets are designed to disintegrate rapidly in the oral cavity and can be administered without water, thereby improving convenience of administration. This formulation offers particular advantages in patients experiencing nausea or vomiting, as well as in those with dysphagia, and may enhance medication adherence in these populations [13, 14]. Advances in formulation technology have recently improved the stability of ODT preparations, and their clinical applicability has expanded, particularly among older individuals and special patient populations [15, 16].

However, changes in formulation may influence the rate and extent of drug absorption. Therefore, pharmacokinetic comparisons with the conventional formulation are essential when developing a new dosage form. In particular, bioequivalence between formulations should be demonstrated using key pharmacokinetic parameters, including maximum plasma concentration (*C*_max_) and the area under the plasma concentration–time curve (AUC), which reflect systemic exposure. Furthermore, because ODT formulations disintegrate in the oral cavity, it is necessary to evaluate the potential for buccal mucosal absorption and to confirm that the overall absorption profile is comparable to that of conventional formulations primarily absorbed in the small intestine.

The objective of this study was to evaluate the pharmacokinetic properties and safety of a single administration of a 20 mg zastaprazan ODT and a conventional tablet in healthy volunteers. In addition, the potential for buccal absorption of zastaprazan following administration of the ODT formulation was evaluated. Through this clinical trial, we aimed to provide evidence supporting the consideration of the zastaprazan ODT as an alternative formulation to the conventional tablet in patient populations who experience difficulty with standard oral dosage forms.

## 2 Materials and Methods

### 2.1 Subjects

This clinical trial was conducted in healthy adults aged 19 years or older. Eligible participants were required to have a body weight of at least 50 kg for males and 45 kg for females, and a body mass index (BMI) between 18.0 and 30.0 kg/m^2^. Subjects were enrolled if they had no clinically significant congenital or chronic diseases and showed no pathological symptoms or abnormal findings on medical history and physical examination.

Individuals who had taken enzyme inducers or inhibitors known to affect drug metabolism within 30 days prior to the first dosing were excluded. Subjects who had received any medication that could potentially influence the study outcomes within 10 days prior to the first dosing were also excluded. In addition, individuals with a history of gastrointestinal resection that could affect drug absorption were not eligible for participation.

Other exclusion criteria included a history of hypersensitivity to the investigational product or any of its components; current treatment with medicinal products containing atazanavir, nelfinavir, or rilpivirine; hereditary disorders such as galactose intolerance, Lapp lactase deficiency, or glucose–galactose malabsorption; and a history of clinically significant psychiatric disorders.

Subjects were recruited from 1 month prior to the first dosing period until the day before dosing. All subjects underwent a physical examination, clinical laboratory assessments, and electrocardiography at the screening visit. Eligibility for participation was determined based on a comprehensive evaluation of these findings, and only those considered suitable were enrolled in the study. Prior to any study‐related procedures, all subjects received a full explanation of the trial and voluntarily provided written informed consent.

The sample size was determined based on the within‐subject variability observed in a previous study of the conventional zastaprazan tablet (JP‐1366‐104; NCT05814809; unpublished data on file, Onconic Therapeutics Inc., Seoul, Republic of Korea), in which the intra‐subject coefficients of variation for *C*_max_ and AUC_t_ were 14.75% and 23.89%, respectively. Based on the higher variability observed for AUC_t_, a within‐subject coefficient of variation of approximately 24% was assumed for the sample size calculation. Assuming a test‐to‐reference ratio of 0.95, 90% power, and a significance level of 0.05, 42 subjects (seven subjects per sequence) were required. Allowing for an anticipated dropout rate of approximately 20%, a total of 54 subjects were planned for enrolment.

### 2.2 Study Design

This clinical trial was conducted as an open‐label, randomized, single‐dose, fasting, six‐sequence, three‐period crossover study. A total of 54 healthy adult subjects were randomly assigned to one of six treatment sequences, each comprising three study periods (Table 1). In each period, subjects received one of three treatments: T1, T2 or R. A dose of 20 mg zastaprazan was used for both the conventional tablet and ODT formulations. This dose was selected because it is the approved clinical dose of zastaprazan in the Republic of Korea, thereby allowing comparison of the pharmacokinetic profiles of the two formulations under clinically relevant dosing conditions.

**TABLE 1:** Study design.

Sequence	Period 1	Washout period	Period 2	Washout period	Period 3	PSV
A	T1	7 days	T2	7 days	R	7 days after Period 3
B	T1		R		T2	
C	R		T1		T2	
D	R		T2		T1	
E	T2		R		T1	
F	T2		T1		R	

Abbreviations: PSV, post study visit; R, Zastaprazan conventional tablet with water; T1, Zastaprazan orally disintegrating tablet without water; T2, Zastaprazan orally disintegrating tablet with water.

The treatments administered in this study were as follows:

- T1: Zastaprazan ODT administered without water
  Subjects first rinsed the oral cavity with 20 mL of water at room temperature and swallowed. One tablet of zastaprazan ODT was then placed on the tongue, allowed to disintegrate completely in the saliva, and subsequently swallowed without additional water.
- T2: Zastaprazan ODT administered with water
  Subjects swallowed one tablet of zastaprazan ODT with 150 mL of water at room temperature.
- R: Zastaprazan conventional tablet administered with water
  Subjects swallowed one conventional tablet of zastaprazan with 150 mL of water at room temperature.

For example, subjects assigned to sequence A received treatments in the order of T1, T2, and R. Specifically, a single dose of T1 (ODT administered without water) was given in Period 1, T2 (ODT administered with water) in Period 2, and R (conventional tablet administered with water) in Period 3. A washout period of 1 week was implemented between each study period. The duration of the washout was determined based on the elimination half‐life of the investigational product and was set at 1 week, corresponding to at least five times the terminal half‐life. The remaining sequence groups were conducted in the same manner according to the randomization schedule presented in Table 1.

The study was conducted at the Biomedical Research Institute, H+ Yangji Hospital, in the Republic of Korea. Subjects were admitted to the clinical unit on the day prior to dosing and were then assigned a randomization number, followed by a standardized evening meal. Fasting was initiated 10 h before dosing. On the following morning, dosing was performed under fasting conditions from 08:00, with administrations separated by at least 1 min. Water intake was restricted for 1 h before and 1 h after dosing. Standardized meals were provided at four and 10 h post‐dose. No concomitant medications were permitted during the study.

Blood samples for pharmacokinetic analysis were collected pre‐dose (0 h) and at 3, 5, 10, 20, 30, 40 and 50 min, and at 1, 1.5, 2, 2.5, 3, 4, 5, 6, 8, 12, 24 and 36 h post‐dose. Blood samples were collected for up to 36 h post‐dose, a duration exceeding three times the reported terminal half‐life of zastaprazan (approximately 7–10 h) [10], to adequately characterize the elimination phase and systemic exposure. Subjects were discharged after completion of the 24‐h pharmacokinetic sampling and returned to the clinic for the 36‐h post‐dose blood collection. At the 36‐h time point, additional diagnostic laboratory tests were performed to assess safety.

This procedure was applied identically in each study period, with a 1‐week washout between periods, and was repeated for a total of three periods. A post study visit was conducted 1 week after completion of all dosing periods. At the post study visit, final safety assessments were performed, including evaluation of adverse events, measurement of vital signs, clinical laboratory tests, and electrocardiography.

This study was approved by the Ministry of Food and Drug Safety of the Republic of Korea and the Institutional Review Board of H+ Yangji Hospital (approval number: HYJ IRB 2024‐10‐024‐002). This study was registered at ClinicalTrials.gov (identifier number: NCT07181538). It was conducted according to the Declaration of Helsinki and the Guidelines for Good Clinical Practice at H+ Yangji Hospital, Republic of Korea.

### 2.3 Bioanalysis

Blood samples were collected into EDTA‐K_2_ vacutainer tubes. The samples were centrifuged at 3000 rpm for 10 min at 4°C. Following centrifugation, approximately 0.5–1 mL aliquots of plasma for analysis and storage were transferred into three polypropylene tubes and stored in a freezer maintained at −70°C until shipment to the bioanalytical laboratory.

Plasma concentrations of zastaprazan and its metabolite, zastaprazan M1, were determined using a validated liquid chromatography–tandem mass spectrometry (LC–MS/MS) method following protein precipitation. The analytical method was validated for selectivity, sensitivity, linearity, accuracy, precision, recovery, carryover, matrix effects, dilution integrity, and stability prior to application to the study samples. The calibration range was 0.5–500 ng/mL, with a lower limit of quantification (LLOQ) of 0.5 ng/mL, and calibration curves showed good linearity, with correlation coefficients (*r*) of ≥ 0.9950. Intra‐ and inter‐batch accuracy was within 85%–115% of the nominal concentration (80%–120% at the LLOQ), and precision was within 15% (20% at the LLOQ). Stability was demonstrated under the evaluated sample handling and storage conditions, with all results meeting the predefined acceptance criteria. For bioanalytical reporting, measurements with a peak area ratio ≤ 20% of that at the LLOQ were reported as not detected (ND), whereas measurements below the LLOQ with a peak area ratio > 20% of that at the LLOQ were reported as below the limit of quantification (BLQ).

### 2.4 Pharmacokinetic Analysis

Pharmacokinetic analyses were performed using Phoenix WinNonlin version 8.4 (Certara L.P.). Non‐compartmental analysis was used to derive the pharmacokinetic parameters of zastaprazan, except for the absorption rate constant (*K*_a_). Actual sampling times for each individual subject were used in the pharmacokinetic calculations, and values were rounded to three decimal places.

For pharmacokinetic analysis, concentrations below the LLOQ at pre‐dose or before *T*_max_ were assigned a value of zero according to the prespecified analysis procedure. Accordingly, values reported as 0.00% in the early post‐dose analysis represent concentrations below the LLOQ that were assigned a value of zero for pharmacokinetic analysis rather than quantified concentrations rounded to 0.00%. For quantifiable pre‐dose concentrations, the pre‐dose concentration was evaluated relative to the corresponding *C*_max_. If the pre‐dose concentration exceeded 5% of *C*_max_, the subject was excluded from all pharmacokinetic analyses. No subjects met this exclusion criterion.

The area under the plasma concentration–time curve from time zero to the last measurable concentration (AUC_t_) was calculated using the linear trapezoidal method. *K*_a_ was estimated separately from the non‐compartmental analysis using compartmental analysis in Phoenix WinNonlin. A one‐compartment model assuming first‐order absorption and elimination was fitted to the plasma concentration–time data using maximum likelihood estimation.

In addition to zastaprazan, its M1 metabolite was evaluated as an exploratory and supportive pharmacokinetic analyte. Zastaprazan is extensively metabolized in the liver, primarily by CYP3A4/3A5, with M1 formed as a Phase I metabolite that has been identified as a major circulating metabolite in humans and reported to possess pharmacological activity [17, 18]. Therefore, zastaprazan M1 was included to provide complementary information on the metabolic disposition of zastaprazan across the different formulations and administration conditions. Accordingly, the systemic exposure and early post‐dose plasma concentration profiles of M1 were evaluated as exploratory and supportive assessments. The pharmacokinetic parameters evaluated are presented in Table 2.

**TABLE 2:** Pharmacokinetic parameters.

Primary endpoints	AUC_t_ and *C*_max_ of Zastaprazan
Secondary endpoints	For zastaprazan: *K*_a_, percentage of concentration relative to *C*_max_, AUC_∞_, AUC_t_/AUC_∞_, *T*_max_, *t*_1/2_, CL/F and *V*_d_/*F* For zastaprazan M1: percentage of concentration relative to *C*_max_, AUC_∞_, AUC_t_/AUC_∞_, *T*_max_, *t*_1/2_

### 2.5 Safety Analysis

The safety analysis was performed for the safety set, which included all 54 subjects who received at least one dose of the study treatment. Adverse events were monitored throughout the study period to evaluate safety. Diagnostic laboratory tests were performed at 36 h after dosing in each period and at the post study visit. Vital signs (blood pressure and pulse rate) were measured at 24 and 36 h post‐dose in each period and again at the post study visit. Electrocardiography was conducted at the post study visit. Adverse events were collected from pre‐dose through the follow‐up visit based on spontaneous reports from the subjects, direct questioning, and investigator observation.

A treatment‐emergent adverse event (TEAE) was defined as an adverse event that occurred or worsened after administration of the study treatment. An adverse drug reaction (ADR) was defined as a TEAE considered related to the study treatment by the investigator. All reported adverse events were summarized according to severity and their relationship to the investigational product, and the clinical significance of any changes in safety parameters was evaluated. All adverse events were followed until complete resolution or stabilization.

### 2.6 Statistical Analysis

Demographic and baseline characteristics were summarized for the full analysis set, which included all subjects who were assigned a randomization number. These variables were analyzed using SAS version 9.4 (SAS Institute Inc., Cary, NC, USA). Descriptive statistical analyses were performed for demographic variables, including sex, age, height, body weight, and body mass index (BMI), as well as other baseline characteristics.

Following bioanalysis of plasma drug concentrations, pharmacokinetic statistical analyses were performed using Phoenix WinNonlin version 8.4 (Certara L.P.) in the per‐protocol set, which included subjects who completed the planned administration of the investigational product and all scheduled pharmacokinetic blood sampling and had evaluable pharmacokinetic data. Pharmacokinetic parameters were summarized descriptively by subject and by treatment group.

For the assessment of bioequivalence, analysis of variance (ANOVA) was conducted on the log‐transformed values of AUC_t_ and *C*_max_ at a significance level of 0.05. The ANOVA model included treatment, period, and sequence as fixed effects and subject nested within sequence as a random effect. The within‐subject coefficient of variation was calculated from the residual mean square error (MSE) of the ANOVA model, which was used as an estimate of the within‐subject variance on the logarithmic scale, using the formula ${\mathrm{CV}}_{w}\left(\%\right)=100\times \sqrt{\exp\left(\mathrm{MSE}\right)-1.}$

Bioequivalence was assessed by calculating the geometric mean ratios and corresponding 90% confidence intervals for zastaprazan ODT administered either without or with water, each compared with the conventional tablet administered with water. Bioequivalence was concluded if the 90% confidence intervals for the geometric mean ratios of AUC_t_ and *C*_max_ were within the predefined acceptance range of 80.00%–125.00%. For zastaprazan M1, geometric mean ratios and corresponding 90% confidence intervals for AUC_t_ and *C*_max_ were also calculated as an exploratory and supportive analysis.

To compare the absorption profiles among the three treatment groups, the mean absorption rate constant (*K*_a_) and median time to maximum concentration (*T*_max_) for zastaprazan were calculated and compared. Furthermore, to assess early systemic exposure following administration of the ODT formulation, plasma concentrations of zastaprazan during the early post‐dose period (3–30 min) were expressed as percentages of *C*_max_ and compared across treatments. The early post‐dose analysis was also performed for M1 as an exploratory and supportive assessment.

Safety assessments were conducted in all subjects who were assigned a randomization number and received at least one dose of the investigational product. Statistical analyses for safety evaluation were performed using SAS version 9.4 (SAS Institute Inc., Cary, NC, USA). Adverse events reported in study subjects were classified and analyzed according to System Organ Class and Preferred Term. Analyses were conducted to describe the incidence of adverse events by severity and by their relationship to the investigational product. Vital signs, clinical laboratory test results, and electrocardiogram (ECG) findings were analyzed using descriptive statistics.

## 3 Results

### 3.1 Subjects

A total of 54 subjects were enrolled in this clinical trial, and all were randomly assigned in equal numbers (nine subjects per sequence) to one of six treatment sequences. Following dosing in period one, three subjects (one each in Sequences A, B, and F) withdrew consent because personal scheduling conflicts prevented them from continuing participation in the study. None of these withdrawals were related to adverse events. Consequently, 51 subjects completed all study periods. Descriptive statistics for demographic characteristics are presented in Table 3. Among the demographic variables, a statistically significant difference between groups was observed for height. However, no significant differences were identified for body weight or body mass index (BMI), suggesting that the observed difference in height was unlikely to have had a meaningful impact on the pharmacokinetic outcomes. No other statistically significant differences in demographic characteristics were observed between groups.

**TABLE 3:** Demographic characteristics of the study subjects.

	Sequence group						*p*
	A	B	C	D	E	F	
Sex (male/female)	9/0	9/0	9/0	9/0	9/0	9/0	NA
Age, years	24.67 (2.18)	22.56 (1.33)	24.00 (2.29)	22.89 (2.85)	24.00 (2.29)	23.33 (2.45)	0.275^b^
Height, cm	171.68 (4.4)	177.97 (4.07)	173.07 (3.47)	177.46 (5.9)	174.53 (6.21)	176.5 (4.36)	0.047^a^
Weight, kg	70.6 (5.52)	76.88 (9.5)	71.04 (7.33)	79.13 (8.87)	73.24 (10.26)	72.54 (5.96)	0.18^a^
BMI, kg/m^2^	23.97 (1.79)	24.23 (2.69)	23.68 (1.88)	25.14 (2.77)	23.99 (2.77)	23.3 (1.85)	0.67^a^

*Note:* Data are presented for the full analysis set. Values are presented as arithmetic mean ± SD.

Abbreviations: NA, not applicable; SD, standard deviation.

^a^ One‐way ANOVA.

^b^ Kruskal–Wallis test.

### 3.2 Pharmacokinetic Assessment

Descriptive statistics for the pharmacokinetic parameters of zastaprazan and its major and active metabolite, zastaprazan M1, following administration of the conventional tablet with water, the ODT without water, and the ODT with water are presented in Table 4. The mean plasma concentration–time profiles of zastaprazan for each treatment are shown in Figure 1, with the corresponding log‐transformed concentration–time profiles presented in Figure 2.

**TABLE 4:** Plasma pharmacokinetic parameters of zastaprazan and its metabolite M1.

Pharmacokinetic parameters	Zastaprazan CT with water (*N* = 51)		Zastaprazan ODT without water (*N* = 51)		Zastaprazan ODT with water (*N* = 51)	
	Mean ± SD	Geometric mean (gCV, %)	Mean ± SD	Geometric mean (gCV, %)	Mean ± SD	Geometric mean (gCV, %)
Zastaprazan						
AUC_t_ (ng × h/mL)	729.29 ± 295.95	681.56 (37.53)	789.64 ± 302.28	737.92 (38.42)	742.68 ± 310.28	695.09 (36.05)
*C*_max_ (ng/mL)	221.77 ± 72.03	210.4 (34.25)	211.59 ± 73.22	199.87 (35.31)	203.02 ± 69.38	191.28 (36.86)
AUC_∞_ (ng × h/mL)	772.77 ± 347.64	715.35 (39.73)	835.43 ± 326.49	778.46 (39.32)	787.41 ± 343.74	732.61 (37.74)
AUC_t_/AUC_∞_ (%)	95.34 ± 3.53		94.86 ± 3.49		94.93 ± 3.20	
*T*_max_ ^a^ (h)	0.667 [0.333–2.500]		0.833 [0.333–2.500]		0.667 [0.333–3.000]	
*t*_1/2_ (h)	9.14 ± 2.80		9.41 ± 2.46		9.39 ± 2.48	
CL/F (L/h)	29.93 ± 11.05		27.55 ± 10.53		28.96 ± 9.37	
*V*_d_/*F* (L)	371.55 ± 121.57		364.16 ± 142.44		375.53 ± 111.23	
*K*_a_	1.41 ± 0.72		1.19 ± 0.70		1.60 ± 0.83	
Zastaprazan M1						
AUC_t_ (ng × h/mL)	451.86 ± 124.92	435.29 (28.51)	471.21 ± 171.99	443.79 (35.67)	457.89 ± 154.31	434.00 (34.22)
*C*_max_ (ng/mL)	97.22 ± 22.92	94.65 (23.74)	91.73 ± 27.67	87.39 (33.36)	89.91 ± 25.92	86.13 (30.92)
AUC_∞_ (ng × h/mL)	497.11 ± 145.38	476.96 (29.92)	527.87 ± 185.84	498.60 (34.95)	503.07 ± 175.99	475.33 (35.06)
AUC_t_/AUC_∞_ (%)	91.51 ± 6.35		89.45 ± 8.05		91.43 ± 4.66	
*T*_max_ ^a^ (h)	0.833 [0.500–2.500]		1.000 [0.500–2.500]		0.833 [0.500–3.000]	
*t*_1/2_ (h)	12.67 ± 5.65		13.87 ± 6.77		12.12 ± 3.36	

*Note:* Data are presented for the per‐protocol set.

Abbreviations: CT, conventional tablet; gCV, geometric coefficient of variation based on treatment‐specific descriptive statistics; ODT, orally disintegrating tablet.

^a^ Data are presented as median [minimum–maximum].

**FIGURE 1:**
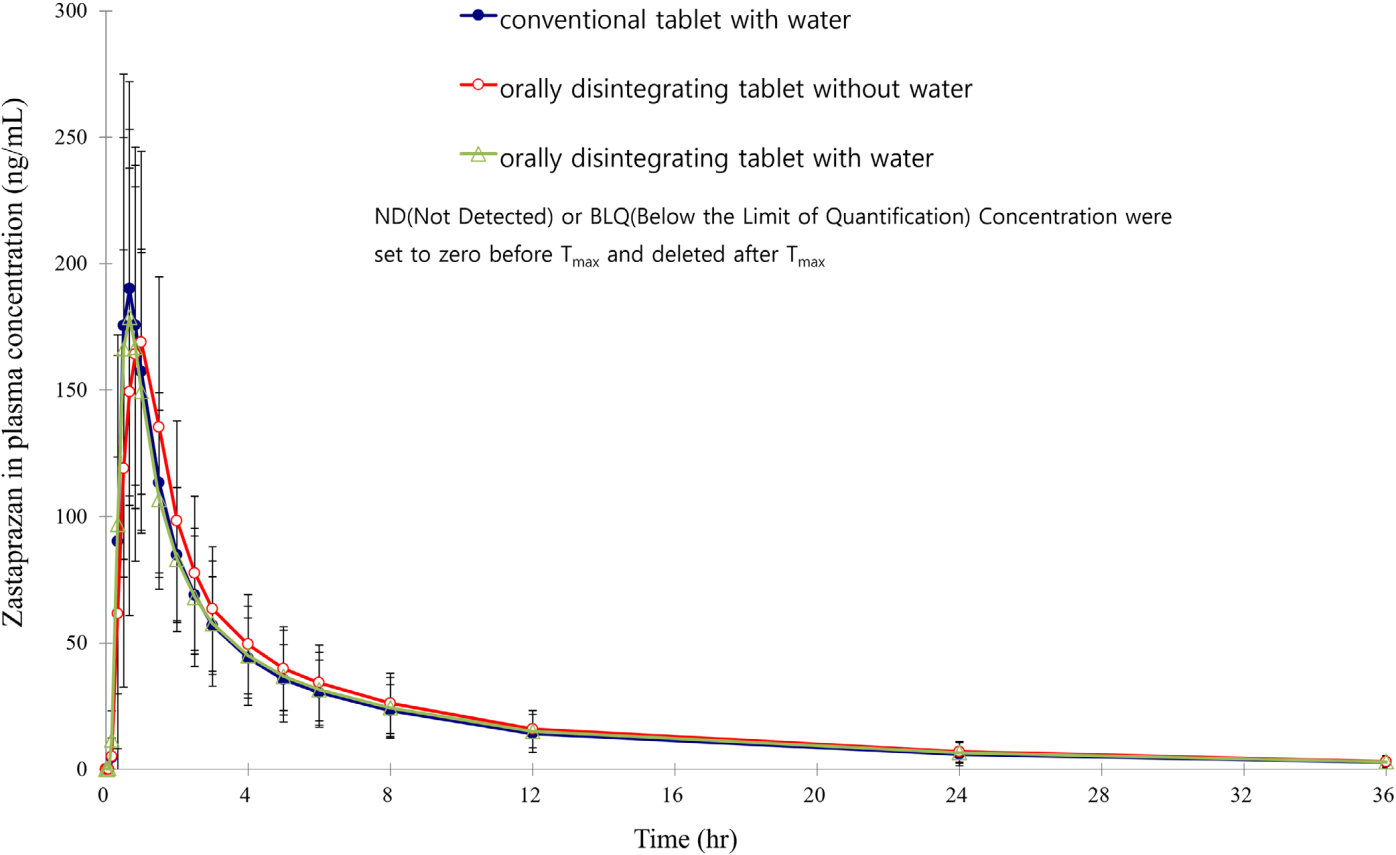
Mean plasma concentration–time profiles of zastaprazan after administration of the conventional tablet and ODT formulations. Data are presented as mean ± SD (*N* = 51). ODT, orally disintegrating tablet; SD, standard deviation.

**FIGURE 2:**
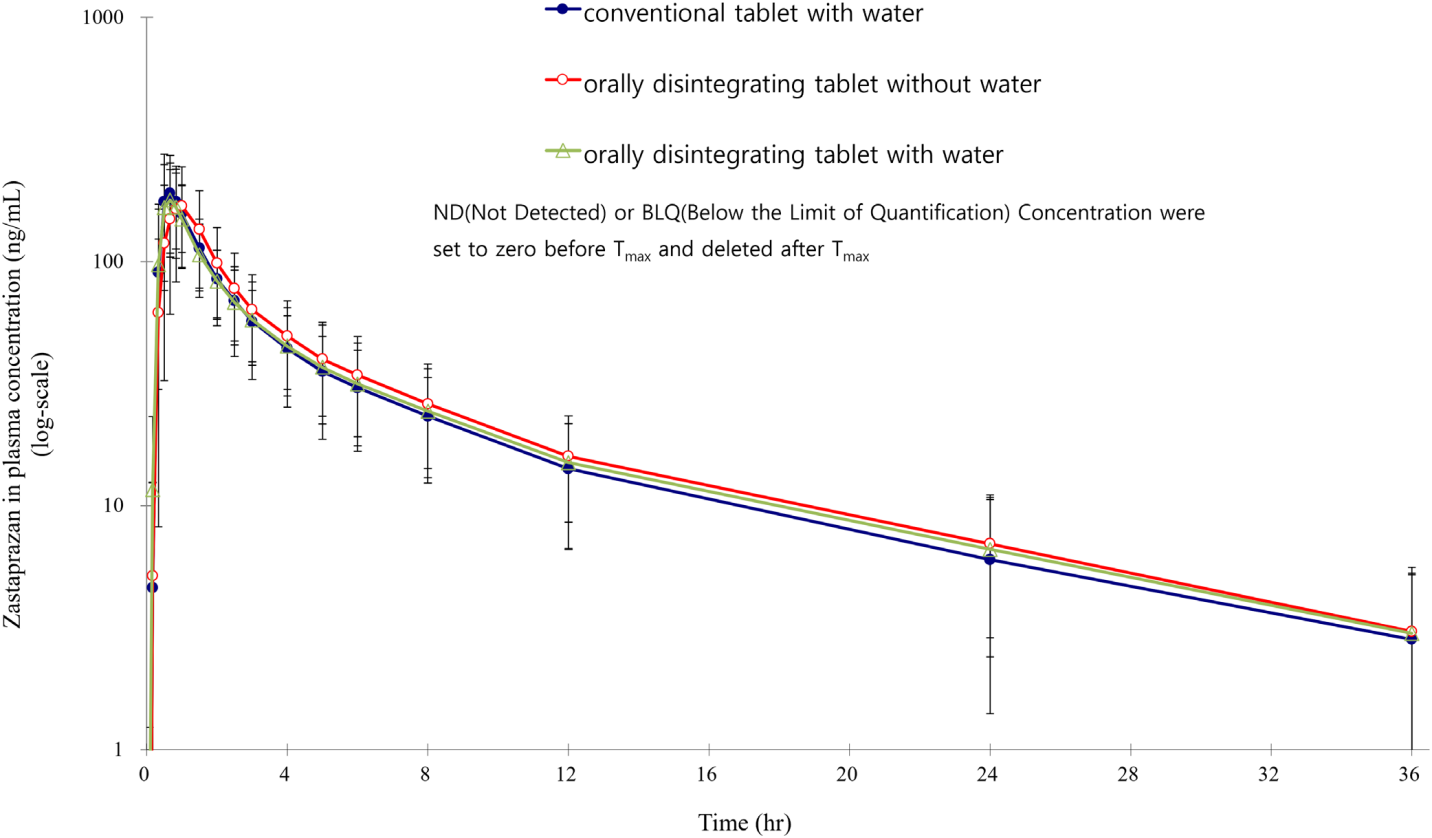
Mean log‐transformed plasma concentration–time profiles of zastaprazan for conventional tablet and ODT formulations. Data are presented as mean ± SD (*N* = 51). ODT, orally disintegrating tablet; SD, standard deviation.

To evaluate bioequivalence between the zastaprazan ODT (administered without water and with water) and the conventional tablet administered with water, geometric mean ratios (GMRs) and corresponding 90% confidence intervals (CIs) for *C*_max_ and AUC_t_ of zastaprazan were calculated (Table 5). When the ODT was administered without water, the GMRs (90% CIs) for *C*_max_ and AUC_t_ compared with the conventional tablet administered with water were 95.05% (88.38%–102.21%) and 108.00% (100.50%–116.06%), respectively. When the ODT was administered with water, the corresponding GMRs (90% CIs) for *C*_max_ and AUC_t_ were 90.85% (84.96%–97.16%) and 101.88% (96.69%–107.36%), respectively. In both comparisons, the 90% CIs for the GMRs of *C*_max_ and AUC_t_ were entirely within the predefined bioequivalence range of 80.00%–125.00%, thereby confirming bioequivalence between the ODT formulation (administered either without or with water) and the conventional tablet administered with water. As a supportive analysis, GMRs and corresponding 90% CIs for *C*_max_ and AUC_t_ of zastaprazan M1 were also calculated. The 90% CIs for both parameters were within 80.00%–125.00% for both ODT administration conditions compared with the conventional tablet, consistent with the findings for zastaprazan.

**TABLE 5:** Geometric mean ratios and 90% confidence intervals for *C*_max_ and AUC_t_ of zastaprazan and M1.

Treatment	Parameter	Geometric mean ratio	90% CI	Geometric means^c^		Intra‐subject CV (%)
				Zastaprazan CT	Zastaprazan ODT	
Zastaprazan	*C*_max_ ^a^	95.05	88.38–102.21	211.03	200.57	22.12
	*C*_max_ ^b^	90.85	84.96–97.16	211.01	191.71	20.40
	AUC_t_ ^a^	108.00	100.50–116.06	682.73	737.36	21.88
	AUC_t_ ^b^	101.88	96.69–107.36	682.743	695.60	15.86
Zastaprazan M1	*C*_max_ ^a^	92.56	87.30–98.14	94.79	87.74	17.72
	*C*_max_ ^b^	90.96	86.00–96.21	94.83	86.26	16.99
	AUC_t_ ^a^	101.91	95.98–108.22	435.97	444.31	18.19
	AUC_t_ ^b^	99.62	95.30–104.15	436.22	434.58	13.43

Abbreviations: CI, confidence interval; CT, conventional tablet; CV, coefficient of variation; ODT, orally disintegrating tablet.

^a^ Zastarazan CT with water—Zastaprazan ODT without water.

^b^ Zastarazan CT with water—Zastaprazan ODT with water.

^c^ Least squares means of geometric.

To compare the absorption profiles among the three treatments, the absorption rate constant (*K*_a_) and *T*_max_ of zastaprazan was calculated (Table 4). The arithmetic mean *K*_a_ values for the conventional tablet administered with water, the ODT administered without water, and the ODT administered with water were 1.41, 1.19 and 1.60, respectively, indicating comparable absorption rates across formulations and modes of administration. And the median *T*_max_ values of zastaprazan were 0.667 h for the conventional tablet administered with water, 0.833 h for the ODT administered without water, and 0.667 h for the ODT administered with water, demonstrating comparable time to peak concentration across treatments. Taken together, these findings suggest that the overall absorption profiles of zastaprazan were comparable between the conventional tablet administered with water and the ODT administered either without or with water.

To assess early systemic exposure following administration of the zastaprazan ODT formulation, plasma concentrations of zastaprazan during the early post‐dose period (3–30 min) were expressed as percentages of *C*_max_ and compared across treatments (Table 6). The corresponding plasma concentration–time profiles during the first 30 min after administration are shown in Figure 3. At 3 min post‐dose, the arithmetic mean percentage of *C*_max_ was 0.00% in all three groups (conventional tablet administered with water, ODT administered without water, and ODT administered with water). At 5 min, the corresponding values were 0.02%, 0.01%, and 0.23%, respectively. At 10 min, the percentages were 1.80%, 2.39%, and 5.71%, respectively, with all treatments remaining below 10% of *C*_max_ during this early time period. At 20 and 30 min post‐dose, plasma concentrations increased in a similar pattern across all three treatment groups.

**TABLE 6:** Percentage of concentration relative to *C*_max_ at early time points.

Pharmacokinetic parameters			Zastaprazan CT with water	Zastaprazan ODT without water	Zastaprazan ODT with water
Zastaprazan	Percentage of concentration relative to *C*_max_ at	3 min	0	0	0
		5 min	0.02 ± 0.08	0.01 ± 0.05	0.23 ± 0.82
		10 min	1.80 ± 3.06	2.39 ± 3.05	5.71 ± 6.02
		20 min	36.61 ± 29.51	28.79 ± 26.91	45.42 ± 26.83
		30 min	75.15 ± 30	55.18 ± 33.37	78.44 ± 26.13
Zastaprazan M1	Percentage of concentration relative to *C*_max_ at	3 min	0	0	0
		5 min	0	0	0.04 ± 0.26
		10 min	0.83 ± 1.67	0.87 ± 1.63	2.73 ± 3.04
		20 min	26.54 ± 23.7	19.87 ± 20.71	33.92 ± 21.59
		30 min	65.88 ± 30.41	45.81 ± 32.40	68.85 ± 28.04

*Note:* Values are presented as arithmetic mean ± SD.

Abbreviations: CT, conventional tablet; ODT, orally disintegrating tablet; SD, standard deviation.

**FIGURE 3:**
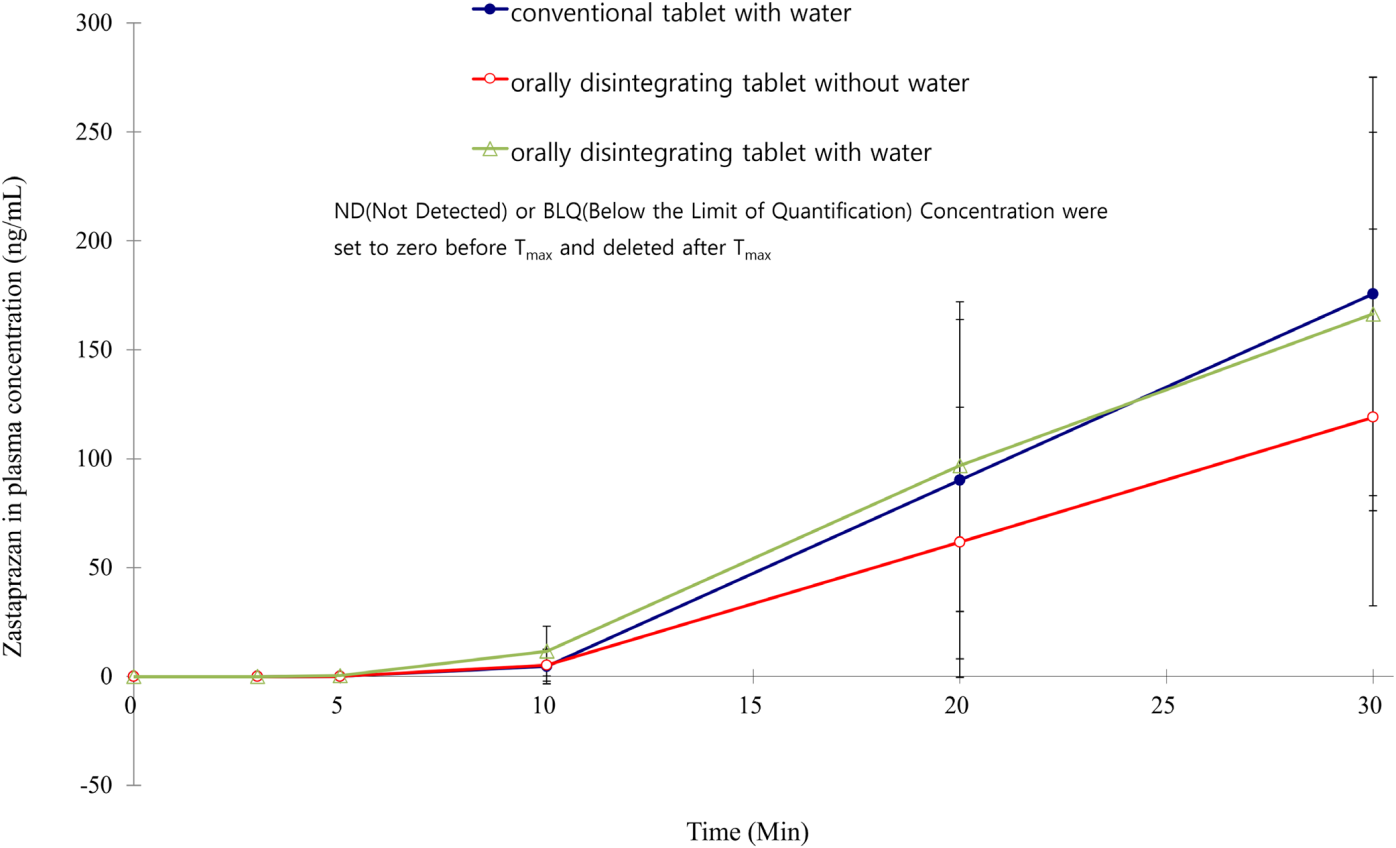
Mean plasma concentration–time profiles of zastaprazan during the first 30 min after administration of the conventional tablet and ODT formulations. Data are presented as mean ± SD (*N* = 51). ODT, orally disintegrating tablet; SD, standard deviation.

As an exploratory and supportive assessment, the same analysis was also performed for zastaprazan M1. The arithmetic mean plasma concentrations expressed as percentages of *C*_max_ were 0.00% in all treatment groups at 3 min post‐dose. At 5 min, the corresponding values were 0.00%, 0.00%, and 0.04%, and at 10 min they were 0.83%, 0.87%, and 2.73%, respectively, remaining below 10% of *C*_max_ across all treatments (Table 6). At 20 and 30 min post‐dose, the increase in plasma concentrations showed a similar pattern among the three treatment groups. Taken together, these early plasma concentration profiles indicate that no apparent additional early systemic exposure was observed following administration of the zastaprazan ODT formulation compared with the conventional tablet.

### 3.3 Safety Assessment

Safety analyses were performed for the safety set, which included all 54 subjects who received at least one dose of the study treatment. Three subjects, one each from Sequences A, B, and F, withdrew consent after completing Period 1 because personal scheduling conflicts prevented them from continuing participation in the study. As these subjects had received the study treatment in Period 1, they were included in the safety analysis. Consequently, the numbers of subjects exposed to zastaprazan ODT without water, ODT with water, and conventional tablet with water were 53, 52, and 51, respectively.

Two treatment‐emergent adverse events (TEAEs) were reported in one of the 51 subjects exposed to conventional tablet with water (Table 7). Based on the temporal relationship, both TEAEs were considered related to the study treatment and were therefore classified as adverse drug reactions (ADRs). The reported adverse events consisted of abnormal laboratory findings, specifically one case of urine red blood cells positive and one case of urine white blood cells positive. Both events were mild in severity, as they were not accompanied by clinical symptoms and resolved without any specific treatment. No adverse events related to vital signs or electrocardiogram findings were observed.

**TABLE 7:** Adverse drug reactions.

System organ class/Preferred term	Zastaprazan CT with water (*N* = 51)	Zastaprazan ODT without water (*N* = 53)	Zastaprazan ODT with water (*N* = 52)
Total	1 (2)	0 (0)	0 (0)
Investigations	1 (2)	0 (0)	0 (0)
Red blood cells urine positive	1 (1)	0 (0)	0 (0)
White blood cells urine positive	1 (1)	0 (0)	0 (0)

*Note:* Data are presented for the safety set. Data are presented as the number of subjects with at least one adverse drug reaction (number of events).

Abbreviations: CT, conventional tablet; ODT, orally disintegrating tablet.

## 4 Discussion

This study compared the pharmacokinetic characteristics of the zastaprazan conventional tablet administered with water and the ODT administered either without water or with water, in order to evaluate potential differences in bioavailability according to formulation. The primary pharmacokinetic parameters were generally comparable across the three treatments, and both ODT administration conditions met the predefined criteria for bioequivalence with the conventional tablet.

In the present study, following single oral administration, plasma concentrations of zastaprazan increased rapidly in all treatment groups, reaching peak levels at approximately 0.667–0.833 h, and subsequently declined with an apparent elimination half‐life of approximately 9 h. The pharmacokinetic profile observed in this study was consistent with previously reported findings for zastaprazan [10].

The 90% confidence intervals for the geometric mean ratios of the primary pharmacokinetic parameters, *C*_max_ and AUC_t_ of zastaprazan, were entirely within the predefined bioequivalence range of 80.00%–125.00% for both the ODT administered without water and the ODT administered with water, compared with the conventional tablet. These findings demonstrate that both ODT administration conditions were individually bioequivalent to the conventional tablet.

As M1 is a major circulating and pharmacologically active metabolite of zastaprazan, its pharmacokinetic findings were evaluated as exploratory and supportive evidence. Systemic exposure to M1, as assessed by *C*_max_ and AUC_t_, was comparable across treatments, with the 90% confidence intervals for the geometric mean ratios falling within the conventional bioequivalence range of 80.00%–125.00%. These findings suggest no formulation‐related difference in M1 exposure and provide supportive evidence for the bioequivalence demonstrated based on the pharmacokinetics of zastaprazan. However, the pharmacological or clinical relevance of M1 exposure was not directly evaluated in this study, and the findings should therefore be interpreted as supportive.

Previous studies have reported that the ODT formulation of tegoprazan, another P‐CAB agent, demonstrated bioequivalence to its conventional tablet formulation with respect to key pharmacokinetic parameters [19]. The results of the present study are consistent with these findings and suggest that the pharmacokinetic characteristics of P‐CAB agents may be preserved despite changes in formulation. Collectively, these data support the feasibility and potential clinical utility of developing ODT formulations within the P‐CAB class.

To further characterize the absorption profiles, the absorption rate constant (*K*_a_) of zastaprazan was calculated and demonstrated comparable values across the three treatments. In addition, the median *T*_max_ values of zastaprazan were comparable among treatments. These results suggest that differences in formulation did not meaningfully influence the absorption characteristics of zastaprazan.

To assess early systemic exposure following administration of the zastaprazan ODT formulation, plasma concentrations during the early post‐dose period (3–30 min) were compared after being expressed as percentages of *C*_max_. Zastaprazan demonstrated very low concentration levels at these early time points across all treatment groups, with no apparent additional early systemic exposure following administration of the ODT formulation compared with the conventional tablet. The same analysis was also performed for zastaprazan M1 as an exploratory and supportive assessment, and similarly low early concentration levels were observed across all treatment groups. Thereafter, the rise in plasma concentrations of both zastaprazan and M1 followed a similar pattern among the three treatments. These findings suggest that, although the zastaprazan ODT disintegrates in the oral cavity, no apparent additional early systemic exposure occurs compared with the conventional tablet.

From a safety perspective, two adverse drug reactions were reported in one subject following administration of the zastaprazan conventional tablet with water. These events consisted of abnormal urinalysis findings, specifically positive results for red blood cells and white blood cells. Based on the temporal relationship, a causal association with the investigational product was considered possible. However, no accompanying clinical symptoms were observed; the events were classified as mild in severity, and they resolved without specific treatment. No clinically meaningful changes in vital signs or electrocardiogram findings were observed in the remaining subjects.

In a previous study evaluating the safety and tolerability of the zastaprazan conventional tablet in healthy adults, mild adverse reactions such as headache and dyspepsia were reported; however, no serious adverse events were observed, and no clinically significant changes in vital signs, electrocardiograms or laboratory parameters were identified [10]. These findings are consistent with the results of the present study, in which the zastaprazan ODT formulation did not reveal any new safety concerns.

Furthermore, previous clinical studies of other agents within the P‐CAB class have reported generally comparable safety profiles. According to earlier reports, P‐CAB agents, including vonoprazan and tegoprazan, demonstrated incidences of adverse events similar to those observed with proton pump inhibitors, with most events being mild to moderate in severity, thereby supporting the overall safety of this drug class [20, 21, 22]. Taken together, the safety findings of the present study for zastaprazan ODT are consistent with those reported for the conventional formulation of zastaprazan as well as for other P‐CAB agents. These results do not suggest the emergence of any new safety concerns associated with the change in formulation.

This study was conducted as a single‐dose trial and evaluated pharmacokinetic characteristics exclusively in healthy adults, which represents a limitation. In clinical practice, ODT formulations are most often intended for individuals who have difficulty swallowing conventional tablets with water. Such patients frequently have underlying conditions, including malignancies or cardio‐cerebrovascular diseases [23, 24]. Therefore, further studies are warranted to investigate the pharmacokinetic properties of the ODT formulation in patient populations rather than in healthy volunteers, in order to better reflect real‐world clinical use.

In conclusion, the zastaprazan ODT administered either without water or with water was bioequivalent to the conventional tablet, with comparable absorption profiles in healthy male subjects. No clinically meaningful safety concerns were identified. These findings support the potential of the ODT as an alternative formulation to the conventional tablet; however, further studies in patients with swallowing difficulties are warranted to establish its clinical utility in the intended patient populations.

## Author Contributions

**Hyung‐Gun Kim:** formal analysis, writing – original draft. **Seunghyun Kang:** investigation, data curation, validation. **Sunyoung Min:** investigation, validation, data curation. **Heejoo Hong:** investigation, validation, data curation. **Moonjung Bae:** conceptualization, methodology, supervision, project administration, writing – review and editing.

## Funding

This work was supported by Onconic Therapeutics Inc., a subsidiary of Jeil Pharmaceutical Co. Ltd., which provided the investigational product. The sponsor had no role in data analysis, interpretation, or manuscript preparation.

## Ethics Statement

This study was approved by the Ministry of Food and Drug Safety of the Republic of Korea and the Institutional Review Board of H+ Yangji Hospital (approval number: HYJ IRB 2024‐10‐024‐002). This study was registered at ClinicalTrials.gov (identifier number: NCT07181538). It was conducted according to the Declaration of Helsinki and the Guidelines for Good Clinical Practice at H+ Yangji Hospital, Republic of Korea. All subjects provided written informed consent prior to screening.

## Conflicts of Interest

The authors declare no conflicts of interest.

## Data Availability

The datasets generated and/or analyzed during the current study are not publicly available due to the inclusion of individual participant data, but are available from the corresponding author upon reasonable request.
